# PROTOCOL: the On Track 2.0 cluster randomized teacher-led intervention to support executive function and self-regulation

**DOI:** 10.3389/fpsyg.2025.1574860

**Published:** 2025-06-24

**Authors:** Anne Marie Kristensen, Steven P. Blurton, Signe Vangkilde

**Affiliations:** Center for Visual Cognition, Department of Psychology, University of Copenhagen, Copenhagen, Denmark

**Keywords:** executive function, self-regulation, curriculum-based intervention, primary education, children, well-being

## Abstract

**Background:**

The top-down cognitive and emotional control skills known as Executive Function (EF) and Self-Regulation (SR) have a large impact on everyday life. As a schoolchild, you are expected to pay attention, wait your turn, follow instructions, solve academic problems and be creative while navigating the social space of peers and teachers. All these abilities draw on EF and SR. Research has pointed to curricular programs as a promising path to build capacity in schoolteachers and provide with further knowledge on ways to support and strengthen these EF and SR skills in their pupils through activities, strategies and reflection tasks. The importance of EF and SR on later life outcomes such as academic performance, career, relationships and risk of crime, has been a motivating factor to develop the 10-session On Track 2.0 intervention as a universal whole-class approach to improve EF and SR in primary school pupils.

**Methods:**

Regular 4th and 5th grade class groups will be invited to participate in this teacher-delivered intervention program to be implemented into regular lessons. In this study schools will be randomly assigned to either intervention or control group as part of a cluster-randomized controlled trial. Psychometric and questionnaire assessing EF, SR and well-being will be administered to children, teachers and parents at three time points.

**Discussion:**

The intervention holds the potential to support and qualify teachers in understanding students’ challenges with EF and SR better and in training these skills during class. A multimodal and multi-informant approach to assessment, in addition to data on teacher adherence and platform use, will aid the insight into the efficacy of the intervention content and delivery.

**Trial registration:**

The study has been registered at Open Science Framework on 22 January 2025.

## 1 Introduction

This study protocol is for a cluster-randomized controlled trial (RCT) of On Track 2.0, a classroom-based teacher-delivered intervention to support and improve executive function for Danish schoolchildren in 4th to 5th grade. The intervention content and design are based on a previous feasibility testing and pilot project of On Track in four Danish primary schools.

Executive Functions (EF) are higher-order functions that refer to conscious top-down cognitive control and enable self-regulation of thoughts, feelings and behavior (Zelazo and Carlson, 2012). EF are applied with a conscious effort when habit and automatic actions are insufficient or inappropriate (Diamond, 2013). Many definitions of EF exist, however there is general agreement that they include the three subdomains of response inhibition (inhibitory control), working memory and cognitive flexibility (or shifting) (Diamond, 2013; Miyake and Friedman, 2012). In combination, these skills enable complex cognitive abilities such as planning, organization, metacognition and self-reflection (Dawson, 2021; Dawson and Guare, 2014). In their study on the unity and diversity of EF, Friedman and Miyake (2017, p. 194) found that the common factor across the three EF subdomains was “the ability to maintain and manage goals and use those goals to bias ongoing processing.” Though this ability is central to inhibition tasks, it is required in all EF tasks (Friedman and Miyake, 2017). This understanding can be conceived as the ability to stay on track and is closely related to other cognitive phenomena such as focused attention and the more colloquial term “concentration.” Focused attention is generally defined as the voluntary act of attending to certain stimuli while ignoring others (Posner and DiGirolamo, 1998), while concentration is defined as the ability to sustain attention and is closely connected to EF such as working memory (Avisar, 2023). Friedman et al. (2007) confirmed the theoretical prediction that attention problems primarily arise due to a deficit in response inhibition. Following from here, the terms concentration and focused attention may be thought of as the foundation for maintaining and managing the above-mentioned goals by supporting and being partly overlapping with inhibition, the common EF factor, thereby enabling successful executive functioning in general.

Related to EF, but not overlapping is self-regulation (Hofmann et al., 2012). SR entails being able to delay instant gratification and stop unwanted impulses in order to pursue long-term goals (Haywood and Lidz, 2006). A pursuit that does not come without the need to regulate and control emotions and frustration. EF have been highlighted as the foundation and prerequisite for successful SR, indicating that efforts to improve EF have the potential to support SR too (Hofmann et al., 2012; Rueda et al., 2005).

Executive function begin to emerge during infancy and develop throughout childhood and have proved to be essential to school success and a range of other life outcomes (Diamond, 2013; Moffitt et al., 2011). Even though EF are not fully mature until adulthood, EF early in life are highly predictive of EF skills later in adolescence and adulthood (Diamond, 2016). In addition, children with poor EF were found to be far less likely to graduate from high school (Diamond and Ling, 2019; Moffitt et al., 2011).

Recent studies suggest that the emotional and cognitive control of adults such as parents and teachers are critical for the caregiving practices involved in children’s EF development (Bardack and Obradović, 2019). A longitudinal study on parental scaffolding practices found that verbal and physical interaction to support the child in solving a ring puzzle task at age 3 were predictive of child EF at age 4 (Hammond et al., 2012). In an observational study, Bardack and Obradović (2019) found that teachers’ display of EF difficulties negatively predicted the assessment of students’ EF skills. These associations underline the potential of designing and providing professional development for teachers focusing on building EF knowledge and scaffolding strategies.

Despite their importance for academic performance and social well-being, EF skills are rarely addressed or listed explicitly in curriculum standards (Dawson, 2021). Systematic reviews and meta-analyses have shown that school-based interventions can improve EF (Diamond and Ling, 2019) and that teacher-student interactions are important for children’s performance in EF (Vandenbroucke et al., 2018). According to Diamond (2016) improving EF may be critical to long-term happiness and life success, and schools and institutions provide a unique place to offer universal efforts and approaches to the benefit of all children in a class group. It is important for interventions to be cognitively engaging, progressively more challenging and personally meaningful to the participants (Diamond and Ling, 2019). However, results from intervention studies are often limited to near-transfer task improvements rather than more global improvements on EF and academic skills across contexts (Melby-Lervåg et al., 2016; Shipstead et al., 2012).

In the autumn of 2022, we piloted the On Track project in 13 classrooms across four randomly selected schools in Zealand, Denmark. The intervention consisted of three researcher-led workshops conducted 2–3 weeks apart in 4th to 6th grade classrooms. The Departmental Ethics Committee approved the project, and participants with parental consent took part in assessments prior to and following the intervention. A combination of the psychometric concentration test d2-R and the Strengths and Difficulties well-being questionnaire was used in addition to a written evaluation interview with closed and open questions. The design did not include a control group making it difficult to draw firm conclusions, but pre-to post-measures improvements exceeded expected retest scores substantially, and the evaluation forms conveyed general contentment with the intervention concept, content and outcome.

The On Track 2.0 intervention program aims to support and train EF in primary school children through educator-led activities integrated into the curriculum. A comprehensive systematic review by Diamond and Ling (2019) highlights that school programs are among the most effective methods for improving EFs. In addition, educator capacity is vital (Muir et al., 2024) and offering further training to teachers significantly increases the likelihood of fostering sustainable changes compared to having activities delivered by outside experts. This approach enhances the opportunity for educators to take ownership of the content and empower them to apply the material in accordance with the strengths and challenges in their specific class groups (Bundsgaard et al., 2018; Muir et al., 2024). In 2023, the amount of Danish early school leavers was higher than 10 years earlier and above the EU average (Eurostat, 2024). According to a recent report, around 15% of 25-year-olds in Denmark have completed only lower secondary education and are not enrolled in an education (Kraka Advisory, 2023). The report concludes that the societal costs associated with the expected loss in earned income is equivalent to about 107 billion Danish kroner (~€14,3 billion) per year group (Kraka Advisory, 2023). The fact that improved EF and SR have a potential positive impact on individual mental health and interpersonal behaviors (Robson et al., 2020) but also high personal and societal costs and justify and encourage further research in interventions to support and train these skills.

On this foundation, we have designed an intervention aimed at improving core EF domains and well-being. Compared to other interventions addressing EF (Diamond and Ling, 2019) On Track 2.0 is novel in its approach as teachers can access and implement the intervention without researcher assistance and interference. The preparation time is minimal, with teacher informational podcasts and pupil activities tapping into core aspects of EF and SR gamified through the story about detective Sofus trying to solve a mystery. Whereas many interventions developed to support EF target pre-school aged children, On Track 2.0 is designed for children in late primary school, addressing a research gap in the (Jacob and Parkinson, 2015) with the potential to help more children to thrive in and outside of school settings.

The goal of improving EF and well-being through On Track 2.0 is explored through the following four research questions (RQs):

RQ1a: Can the On Track 2.0 intervention improve EF in Danish 4th and 5th grade pupils from pre-to post-intervention assessment?

RQ1b: Is the potential EF improvement of the On Track 2.0 intervention sustained until or increased at the follow-up assessment?

RQ2a: Can the On Track 2.0 intervention improve child-reported well-being in Danish 4th and 5th grade pupils from pre-to post-intervention assessment?

RQ2b: Is a potential improvement in well-being sustained at the follow-up assessment?

In addition, we will explore the following research questions:

RQ3: Do mechanisms such as age, gender, dosage or baseline performance affect either outcome?

RQ4: Does the intervention affect other variables measured such as subdomains of the psychometric tasks (d2-R and Stop Signal Task) or parent-and teacher-reported EF and well-being?

The following sections detail the intervention design, content, and assessment methods and strategies employed to ensure adherence and minimize bias.

On Track 2.0 is a 10-week cluster-randomized, teacher-led intervention integrated into regular classroom teaching. The target group is 4th and 5th grade pupils at standard Danish primary schools. Following the recommendations by Campbell et al. (2000) for complex health interventions, the study consists of four distinct phases. Based on a solid theoretical foundation, Phase I involves designing, modeling and qualitatively testing the content and details of the intervention. During this phase, editors at publishing company Forstå, who provide freely available online teaching materials to schools, assist practically with designing and setting up the intervention content on the online platform while offering didactical feedback.

Phase II entails an exploratory trial, which will include a design and feasibility testing involving a small number of classrooms. Different variations of the content may be tested and focus group interviews with teachers and pupils will inform the adaptation of the material prior to the RCT.

Following this stage, we will recruit schools and implement the definitive intervention in a cluster RCT. In Phase III intervention and teaching-as-usual control groups will be randomized on the school level to avoid contamination between class groups within schools. Control groups will be offered access to the intervention materials as soon as all assessments are completed in their group. For practical reasons, we plan to run the intervention in two waves (Wave 1 in the spring, and Wave 2 in the autumn), testing intervention and control groups in parallel.

Phase IV involves the transition from testing the materials to making them freely available to all teachers and promoting implementation in schools across the country. Results will be disseminated in academic journals but also to teachers and practitioners through targeted publications and presentations.

Many research studies have found that the continued implementation of evidence-based mental health interventions often face common barriers such as lack of teacher support, difficulties obtaining materials, and finding the time amongst many other obligations and priorities (Locke et al., 2015). To avoid similar challenges, more evidence-based teaching materials should be made freely available and easy to access and implement into standard classroom settings without the need to acquire new equipment (Banerjee et al., 2023).

## 2 Methods

### 2.1 Setting

The intervention is designed to be delivered at Danish primary schools (public, private, or free schools) by teachers to their regular class groups. With the Danish inclusion policy, most class groups will have children with special needs. However, in the pursuit of creating comparable treatment groups, whole class groups for children in special education or with significant learning disabilities will not be included. After the assignment to intervention and control groups, the teachers involved will attend separate online information meetings. They will then get access to the teaching materials on a digital platform including podcasts and activities as explicated below. Data will be collected at the schools in an environment known to the children to improve validity and to minimize the inconvenience for participants.

### 2.2 Study population and eligibility criteria

Teachers of class groups from 4th and 5th grade will be invited to take part (children aged 9–12 years). As teachers in Denmark often work in teams, whole year groups are encouraged to take part, and the material includes topics designed to be discussed between teachers during subject-specific team meetings (The Danish Evaluation Institute, 2021). Teachers agree to partake on behalf of their entire class group, and all children in an intervention class group take part in the intervention. Children with mild learning disorders or limited Danish proficiencies with parental consent to participate in assessments will be supported by researchers when completing questionnaires.

### 2.3 Intervention and comparator

The 10-week teacher-led intervention is structured into five modules, which are made up by the three central subcomponents of EF (response inhibition, working memory and cognitive flexibility) (Friedman and Miyake, 2017) and the related skills of self-regulation and metacognition (Buttelmann and Karbach, 2017; Hofmann et al., 2012). Whereas the first four modules are based on foundational EF skills, metacognition may be perceived as an advanced skill developing later in childhood (Dawson, 2021), which is why this skill is the last to be explored during the intervention. Each of these five pillars are to be explored and implemented into regular classes during two school weeks (Table 1). Participating teachers get access to the material on a digital platform made available by publishing company Forstå (Forstå-Gratis undervisningsforløb og undervisningsmaterialer, n.d.).

**Table 1:** Content and narrative of the five intervention modules.

	Module 1: Impulse control		Module 2: Working Memory		Module 3: Cognitive flexibility		Module 4: Self-regulation		Module 5: Metacognition	
	Week 1	Week 2	Week 3	Week 4	Week 5	Week 6	Week 7	Week 8	Week 9	Week 10
Narrative	Presentation of Sofus and the circumstances		Examine the crime scene		Explore suspects (different angles and motives)		The situation is coming to a head–stay calm!		The mystery culminates	

The teaching materials are developed by authors AMK and SV and consist of knowledge and research-based suggestions for teachers and an educational narrative with associated activities for the pupils.

From a pupil perspective the material revolves around the detective Sofus. When they first meet Sofus, someone has rummaged through his office, and he needs the help of the class group to solve the mystery ahead of him. In the narrative, someone shuts down the internet, which adds elements of technology education and reflections on digital distraction, privacy, and climate. The story works as a common third for pupils and teachers to work with, with the obvious purpose of helping Sofus and the indirect purpose of transferring and internalizing the strategies that he needs and uses to themselves.

In Table 2, the activities connected to each EF skill are described briefly. The modules are available to teachers in a sequential order progressing from more basic EF skills and finishing with metacognition which develops later in childhood and can be conceived as a more advanced EF (Dawson, 2021).

**Table 2:** Progressive intervention activities by executive function and SOFUS skills.

EF skill	SOFUS skill	Week	Activities
Impulse control	Strategies Make a plan Stop Stay calm	1	Sofus’ Office: Gather clues and leads (identify potentially relevant items). Simon Says (variation): React only to instructions given in a certain voice or manner; ignore others.
		2	Double Circles: Discuss who needs the internet, what do you like to do when online, and what would happen if the internet was shut down? Forbidden Words: Explain certain words to a partner for them to guess without mentioning three descriptor words printed in red. In round two, use only mime.
Working memory	Overview Use memos Follow your plan Organize	3	Who’s Mar? Come up with as many names as possible starting with “Mar.” The Library: Sort leads and clues into categories to create an overview.
		4	Using Memos: Look at the pictures or a selection of things and recall them after they are covered. Code Language: Try to decode secret messages using a symbol key and learn some of the symbols by heart.
Cognitive flexibility	Flexibility In other eyes Adjusting Get ideas	5	Plan the Route: Plan Sofus’ route to visit suspects taking the order of important events into account. Looking into Suspects: Identify who the suspects are based on Sofus’ notes?
		6	In the Eyes of the Suspects: Divide the class into interviewers and interviewees; interviewees role-play as one of the two suspects. Alibies: Work out what the evidence says about the alibies of the suspects by flipping the text in your head.
Self-regulation	Perseverance Patience Practice makes perfect Determination	7	Calming Down Sofus: Discuss strategies for managing anger using Sofus’ frustration as a case. What helps you to calm down when angry? Thought Channels: Pretend that you can shift your thoughts with a controller. Talk about three elements in the picture one at a time and shift when asked to.
		8	Help Sofus Recap and Review: Reflect on what has happened so far; have any leads or clues been left out and need following up. Sofus’ Boss: Use her plane ticket to work out whether she has an alibi.
Meta-cognition	Self-reflection Who am I? What is going well? What is hard?	9	Self-Reflection: What things are currently going well and which things you would like to become better at. The Negotiation: What agreement can Sofus make with his colleagues about the internet? How can they use it in a responsible, controlled way?
		10	What would the others think: When seeing things from other characters’ points of view, what might they think about the deal made last week? Cleaner’s room: Examine the notes and review all the strategies you have developed throughout the intervention period.

Sofus is a relatively common Danish name, but in this context Sofus also serves as an acronym linking directly to the intervention’s main cognitive concepts as presented in Table 3 below:

**Table 3:** The Sofus acronym and related cognitive concepts in Danish and English linked to executive functions (EF).

	**S**	**O**	**F**	**U**	**S**
Danish word	Strategier	Overblik	Fleksibilitet	Udholdenhed	Selvrefleksion
English word	Strategies	Overview	Flexibility	Perseverance	Self-reflection
EF	Inhibition	Working memory	Cognitive flexibility	Self-regulation	Metacognition

These five skills are central to the activities and the narrative. During each of the five modules, the pupils complete a selection of activities that engage the module skill in focus, resulting in leads that aid them in solving the mystery ahead. Each week there are multiple activities available, and teachers are encouraged to implement at least one activity each week. Customized illustrations were created to support and accompany each module.

The digital platform will contain all instruction material and teaching content. The topic of each of the five pillars will be introduced through 6–10-min explanatory podcasts, featuring research experts and practitioners from within the field.

In the project a teaching-as-usual control group will be included. This group will be offered to access the On Track teaching materials as soon as assessments are completed, before the materials are made publicly available.

Both groups will be offered a post-intervention presentation on the development and common challenges to EF and SR skills, and an explanation of the average class-level progress during the intervention period based on the aggregated data collected for their class.

### 2.4 Intervention compliance

To monitor adherence to the program, teachers in the intervention group will receive a brief electronic survey every 2 weeks. In this survey they answer a small number of questions about which exercises they used in class and how well they worked. In addition, we can access user time use data through the publisher and send teachers a text reminder to encourage engagement if they have not accessed the platform within the last week.

### 2.5 General description of assessments

Researchers and research assistants will conduct assessments in the participating schools at three time points; t_0_: 1–2 weeks prior to the intervention period, t_1_: 1–2 weeks after the intervention period, and t_2_: 4 months after the intervention period (follow-up assessment). During assessments, pupils will complete psychometric tasks and questionnaires on iPads in a room where there is space between them and minimal visual and auditory distractions. Parents and teachers will receive secure links to their questionnaires, which they can complete privately at home.

Assessments will be conducted on-site at the schools involved in the project, and intervention and control groups will be tested in parallel. Whenever possible, research assistants who are blinded to the group allocation will conduct the data collection to minimize the potential risk of bias (Higgins and Cochrane Collaboration, 2020). For practical reasons, it will not be possible to blind main researchers and participating teachers to group allocation. However, because regular teachers will implement the materials into their own classes over 10 weeks, the risk of Hawthorne effects is expected to be smaller compared to researcher-led interventions (Murnane and Willett, 2011). To enhance study validity and reliability, more than one mode of assessment is used (multi-modal), multiple informants report on each child (multi-informant) and these informants represent different contexts from the child’s everyday life (multi-setting) (Sparrow, 2010).

To ensure ethical research practices during assessments, researchers and research assistants will talk to children in a developmentally appropriate language, thereby obtaining informed consent in a manner aligned with their understanding and developmental level (Field Marilyn et al., 2004).

### 2.6 Psychometric tasks

All tests will be administered by researchers and research assistants and completed on iPads (10th generation, 10.9-inch) at each school.

*d2-R* is a digital concentration and attention test (Hogrefe, n.d.) comprising 14 20-s trials, each containing 60 items (letters d or p with 0, 1, or 2 dashes above or below the letter). The participant must press as many targets as possible (d’s with two dashes) and ignore all distractors (all other letter/dash combinations). The d2-R score represents processing speed and constitutes the primary outcome measure. Due to the close relationship between the focused attention skills needed for this task and executive functions such as working memory and inhibition, the d2-R is used as a proxy measure for EF in the context of this study.

*Stop Signal Task (SST)* is a task to assess response inhibition. The participant has to inhibit their response to press the screen when occasionally presented with a stop signal amongst primarily go signals (Logan, 2015; Logan et al., 1984). We will include individual stop signal reaction times as a secondary outcome measure in our exploratory analyses.

### 2.7 Questionnaires

*Demographic information:* Information on child age and sex will be collected at baseline through parent consent forms.

*Behavior Rating Inventory of Executive Function, Second Edition (BRIEF-2) Screening form* will be included in parent-and teacher reports which cover the age range of 5–18 years. This abbreviated version of the BRIEF-2 questionnaire (PARinc, n.d.) assesses EF through 12 items that are answered in a three-point Likert format with the response options “Never,” “Sometimes” and “Often” (Gioia et al., 2015). Based on Danish norm data from a group of children with typical development, there is good internal reliability with the parent and teacher report reaching a Cronbach’s *α* coefficient of 0.84 and 0.93, respectively (Ziska, 2024). The BRIEF-2 Screening form must be interpreted with caution when used for individual screening scores, as it has been recommended for research rather than as a diagnostic tool in clinical practice (Ziska, 2024).

*KIDSCREEN-10:* 10 items measure global Health-Related Quality of Life for monitoring and screening purposes (Ravens-Sieberer et al., 2010). The questionnaire will be included in self-report (age 8–18) and parent-report versions. KIDSCREEN-10 is a shorter version of the KIDSCREEN-52 which takes about 5 min to complete (kidscreen.org, n.d.). Despite certain criticisms relating to the psychometric properties of the Danish version of the KIDSCREEN-10 (Nielsen et al., 2023), it was selected over other well-being questionnaires such as the Strengths and Difficulties Questionnaire (Goodman, 2001) to obtain self-report measures.

*Activity Perception Questionnaire* consists of 25 items rated on a 6-point Likert scale and is adapted to this context based on the survey by Deci et al. (1994). Selected items will be used to evaluate and monitor teacher adherence and intervention fidelity, motivation and perception of intervention content (Beaven et al., 2017).

### 2.8 Participant timeline

**Figure 1:**
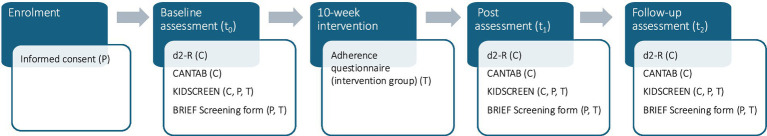
Intervention and assessment timeline.

The intervention and assessment timeline for children (C), parents (P) and teachers (T) is presented in Figure 1:

Baseline assessments take place immediately before the intervention (t_0_) and post assessments immediately after (t_1_) as mentioned above. Follow-up assessments (t_2_) take place 3-4 months after intervention completion.

### 2.9 Analytic sample and sample size

Power calculations are based on the effect sizes reported in comparable intervention studies using the d2-R Attention test as the primary outcome. Schmidt et al. (2015) estimated the effect size needed to achieve statistical significance to reject the null hypothesis based on studies by Gallotta et al. (2012, 2015) using the d2 paper and pencil test. Schmidt et al. (2015, p. 435) present an “*a priori* power analysis with power (1 – beta error probability) = 0.80, alpha error probability = 0.05, effect size *f* = 0.10 [*d* = 0.20], number of groups = 2, number of measurements = 3, and correlation between the repeated measures *r* = 0.75.” This power calculation resulted in a sample size of *N* = 82 students. Schmidt et al. (2015) themselves achieved a medium effect size of *f* = 0.404 (*d* = 0.81). Based on these findings, we chose a minimal detectable effect size of *d* = 0.4, aiming to strike a balance between detectability and feasibility (Dong and Maynard, 2013).

Given that each school will contribute 1 year group consisting of two to three class groups into the intervention and control groups, we must consider the effects of nesting and intra-class correlation coefficient (ICC). The ICC can be defined as follows:


$$\mathrm{ICC}=\frac{\sigma_{\text{class}}^{2}}{\sigma_{\text{class}}^{2}+\sigma_{\text{school}}^{2}+\sigma_{\text{residual}}^{2}}$$

The ICC can be interpreted as the share of the total variance accounted for by the variance between clusters and expresses the strength of the similarity of results within clusters compared with the similarity between clusters (Dreyhaupt et al., 2017).

Based on our pilot project data using the d2-R test on 79 children from eight classrooms in three schools, we ran a linear mixed model in R and found ICC_school_ = 0.00 and an ICC_class_ 0.09. Because this ICC is relatively unreliable, as it is based on a small number of clusters, we also refer to Murnane and Willett (2011), who highlighted that a medium sized ICC is *ρ* = 0.09. The advantage of having based the power calculations on our pilot data is that these children are similar in age and come from a mixed socio-economic background.

According to national statistics, there are 21 children per class on average (Ministry of Children and Education, 2024a), and based on the pilot test we assume that we will get parental consent to take part in tests from around 75% of a class group. We expect an additional attrition across t_1_ and t_2_ of 10% and exclude participants in accordance with the d2-R manual (Hogrefe, n.d.). Thus, we calculate the statistical power based on an effective class group size of 14 individuals.

To find the sample size required to detect a minimum effect size of *d* = 0.4, we used PowerUp (Dong and Maynard, 2013). In addition to the ICC values mentioned above, we calculated the minimum number of schools (level 3 units) to be recruited, by including the proportion of group-level variance explained by covariates such as baseline performance, age and gender (level 1) and school size, grade point average and absence (level 3) (Ministry of Children and Education, 2024b). Including these covariates reduces the group-level residual variance and thereby increases the statistical power (Murnane and Willett, 2011).

As shown in Table 4, we need a total of 12 schools to achieve sufficient power to detect an effect of size *d* = 0.4. To account for attrition at the school level, we plan to recruit a total of 16 schools that will be randomly assigned to intervention and wait-list control groups.

**Table 4:** PowerUp calculation of sample size including assumptions and comments.

Factors and Assumptions	Values	Comments
MRES = MDES	0.40	Minimum Relevant Effect Size = Minimum Detectable Effect Size
Alpha level (α)	0.05	Probability of Type I error
Two-tailed or One-tailed test?	2	
Power (1-β)	0.80	Statistical power (1–probability of Type II error)
Rho_3_ (ICC_3_)	0.00	Proportion of variance in outcome between Level 3 units: V3/(V1 + V2 + V3)
Rho_2_ (ICC_2_)	0.09	Proportion of variance between Level 2 units: V2/(V1 + V2 + V3)
*P*	0.50	Proportion of Level-3 units randomized to treatment
*R*_1_^2^	0.60	Proportion of variance in Level 1 outcome explained by the Level 1 covariates
*R*_2_^2^	0.20	Proportion of variance in Level 2 outcome explained by the Level 2 covariates
*R*_3_^2^	0.10	Proportion of variance in Level 3 outcome explained by the Level 3 covariates
g_3_*	1	Number of Level 3 covariates
n (Average sample size for Level 1)	14	Mean number of Level 1 units per Level 2 unit (harmonic mean recommended)
J (Average sample size for Level 2)	2	Mean number of Level 2 units per Level 3 unit (harmonic mean recommended)
M (Multiplier)	3.15	Automatically computed
K (Sample Size [# of Level 3 units])	**12**	Number of Level 3 clusters needed for given MDES.

Light yellow values are commonly adopted levels and are preset (Dong and Maynard, 2013). The parametres in dark yellow are specified to the On Track 2.0 study.

### 2.10 Recruitment and randomization

Schools will be invited to participate through a combination of e-mails, newsletters and social media to teachers and school principals across the country. Representativeness of the participants is crucial to the external validity of the intervention (Glasgow et al., 1999). We plan to recruit schools from diverse municipalities, which will be randomized into intervention and teaching-as-usual waiting list control groups using a computer-generated random sequence unavailable to those enrolling and assigning schools. All recruited schools will be randomly allocated to avoid skewing results by including a highly motivated experimental group (Conrad, 1987). Ideally, to minimize bias, both program providers (teachers) and pupils should be blinded to whether they are in a treatment or control group (Schulz and Grimes, 2002). While complete blinding is challenging to achieve in psychological intervention studies, every effort well be made to maintain objectivity in the process. When including a waitlist control group, blinding teachers is not feasible to achieve. However, data collection and analyses will be conducted by different people, and analyses will be conducted on anonymized data by a researcher, who is blinded to group membership.

### 2.11 Data management and analysis

All data collected during this project will be stored on secure servers provided by the University of Copenhagen. Data handling will strictly adhere to GDPR regulations. A data management risk assessment has been completed. Data management agreements with companies used for data collection (Forstå, Hogrefe, and SurveyXact) have been made.

Data analysis will commence once all data has been collected.

### 2.12 Ethical considerations

The Ethics Committee at the Department of Psychology, University of Copenhagen, approved the project (Approval No. IP-EC-25102024-1). When a class is enrolled in the study, parents receive information about the project and the assessments. Only children with written parental consent are included in assessments.

### 2.13 Data analysis

Statistical analyses will be conducted to examine the effects of the On Track 2.0 intervention. Descriptive analyses will be conducted of pupil, classroom, and school characteristics and of teacher adherence to the intervention.

The primary outcome, the d2-R concentration scores, will be analyzed in a three-level hierarchical linear model (West et al., 2022), with Age, Sex, and Performance at baseline included as covariates. The secondary outcome, well-being, will be analyzed in a similar fashion. Research question RQ1a will be tested with a comparison of the primary outcome variable between intervention group and control group measured at post-intervention. Research question RQ1b will be addressed with the same group comparison but based on the measurement of the primary outcome at follow-up. Research questions RQ2a and RQ2b involve the same group comparisons at post-intervention and follow-up, tested on the secondary outcome variable (child-reported well-being). We plan to test for the effect of the second level (classes within schools) and remove it from the model, if nested model comparisons do not significantly improve the model fit of the three-level model.

Exploratory analyses connected to RQ3 and RQ4 will include well-being questionnaires completed by parents and teachers, subdomains of d2-R and stop signal task performance, the intervention dosage if possible (e.g., number of lessons completed), and additional covariates.

### 2.14 Validity checks

The dynamic and varying nature of school week schedules – often interrupted by, e.g., field trips, thematic weeks, or class conflicts – poses challenges to maintaining consistency across intervention and control groups. Even though this is expected to be the case for both groups, adherence and consistency across teachers will pose a problem to the internal and external validity (Darling et al., 2021). However, the fact that teachers can also adapt the material to their own practice may enhance the likelihood that they will actively use the materials and transfer some of the principles to their own practice (Bundsgaard et al., 2018). The short fidelity questionnaires in addition to platform usage data will provide important insight into teacher adherence, enabling dose–response evaluations. If possible, platform usage logs will be included in analyses too.

Attrition poses another risk to the internal and external validity, particularly if differential drop-out occurs between groups. To mitigate and account for this, intention-to-treat analyses and imputations according to the missingness of the data will be employed.

## 3 Discussion

Based on the On Track feasibility study, the On Track 2.0 intervention offers a promising approach to support and qualify teachers in better understanding students’ challenges with executive function (EF) and self-regulation (SR), and in training these skills within everyday classroom teaching. The intervention is designed to build teachers capacity with the aim of sustaining approaches and methods beyond the intervention period. To promote the transfer of skills across contexts (Aarkrog, 2010), strategies and activities are introduced gradually over 10 weeks and revisited through reflection by both teachers and pupils. The decision to design and implement a teacher-delivered, adaptable intervention inevitably introduces some variability in adherence and implementation across the intervention groups (Darling et al., 2021). However, ensuring that interventions are meaningful and relevant to the teachers involved is essential if they are to be adopted and sustained in practice (Muir, 2024). Conducting intervention studies in real-world school settings is inherently complex. The validity and reliability of findings are influenced by numerous contextual variables beyond researchers’ control – such as staff absences, unplanned extra-curricular events, teacher motivation, and class dynamics (Wheatley et al., 2020). As such, conclusions drawn from the collected data must interpreted with caution, as these contextual factors may add to statistically significant, partial, or null effects that do not fully reflect the intervention’s potential. Importantly, even if task performance improves, translating those gains into broader academic achievement and well-being outcomes remains challenging. Such transfer often requires continued teacher engagement, reinforcement, and scaffolding beyond a structured intervention period (Gunzenhauser and Nückles, 2021).

Including questionnaires completed by teachers imposes a considerable time burden to the teachers involved. To minimize potential bias, each teacher will receive a randomized list of the pupils in their class who have parental consent to take part in assessments. The teachers are asked to assess the pupils in the order specified in the list to ensure that their decisions on who to assess are not affected by time constraints or subjective preferences if they cannot complete questionnaires on everyone.

In general, limitations associated with assessing EF are well established; correlations between psychometric tests and questionnaire ratings are modest at best, and each approach comes with distinct strengths and weaknesses (Pino Muñoz and Arán Filippetti, 2019; Toplak et al., 2013). While widely used, the BRIEF-2 has been criticized for poor validity of its subscales, which often fail to differentiate between distinct executive profiles (Jacobson et al., 2020; Lace et al., 2022). Furthermore, associations between scores on EF rating scales and academic performance show mixed results (McAuley et al., 2010; Pino Muñoz and Arán Filippetti, 2019) with a risk of cultural biases (Thorell et al., 2013). Given the time constraints of teachers, administering the full BRIEF-2 questionnaire for all pupils with parental consent at three time points was deemed unfeasible. In contrast, the 12-item BRIEF-2 Screening Form offers a more pragmatic solution, though it may be subject to some ceiling effects in our sample of children with predominantly typical development. While brief, it allows us to capture a broad indication of EF functioning. Moreover, studies indicate that performance-based tests and questionnaires assess two different underlying processes of EF (Lace et al., 2022; Toplak et al., 2013). From this perspective, employing both approaches in this study is considered a clear methodological advantage.

To address the challenge of measuring EF and SR with sufficient ecological validity (Soto et al., 2020; Souissi et al., 2022), we employ a multimodal, multi-informant approach. Laboratory-style, performance tests, conducted in quiet and structured conditions, may not fully capture children’s real-world EF capacities amid an everyday life full of sounds, classmates, family and potential distractions (McAuley et al., 2010). However, striking the right balance between including the minimal number of tests necessary to avoid fatigue but enough measures to paint the full picture is a challenge. To balance these concerns, we have prioritized brief, concise, and developmentally appropriate tests and questionnaires that can be administered effectively in schools.

A limitation of the this study is that the three core EF components (inhibition, working memory, and cognitive flexibility) are not assessed separately with psychometric tools. We try to accommodate this by targeting inhibition, the common and underlying component according to Friedman and Miyake (2017). For well-being, we chose the KIDSCREEN-10, a brief, validated, and accessible (also in Danish) measure suitable for the age group. Using more targeted questionnaires such as child-reported attention control or academic well-being could offer other advantages, but we found no validated Danish tools of appropriate length and suitability for this population.

Exploring the potential of interventions to enhance executive functions (EF) in children beyond the pre-school years remains a critical and underexplored domain in educational research (Jacob et al., 2022). This focus also aligns with the OECD’s recommendation that intervention programs for children should target both cognitive, social, and emotional skills (OECD, 2015). If the current project supports the efficacy of the On Track 2.0 program, we envision that the program can be developed and scaled to be implemented for children in early primary school, in secondary school pupils and even in special education settings. A successful scaling would rely on having assessed and addressed important contextual moderators and implementation fidelity but offers interesting potentials for embedding EF across school curricula, so all students can benefit. Cost-effective programs that build capacity in teachers and help them to support and strengthen EF skills in their students through engaging and enjoyable activities are not only relevant today but will continue to be so in the future. Given the increasing demands of 21st-century classrooms—such as managing digital distractions and promoting autonomous learning—it is more important than ever to equip teachers to support and develop creativity, focus, and critical thinking among their pupils (Meltzer, 2018; Vincent-Lancrin et al., 2019).
